# Valorization of Crispy Fried Shrimp Leg By‐Products in the Development of Nutritionally Enhanced Extruded Snack Products

**DOI:** 10.1155/ijfo/9953054

**Published:** 2026-11-10

**Authors:** Riantong Singanusong, Kanyarat Banyam, Jiratchaya Judphol, Saowaluk Rungchang

**Affiliations:** ^1^ Department of Agro-Industry Faculty of Agriculture Natural Resources and Environment Naresuan University Phitsanulok Thailand nu.ac.th; ^2^ School of Agro-Industry Faculty of Agro-Industry Chiang Mai University Chiang Mai Thailand cmu.ac.th

**Keywords:** calcium-rich ingredient, corn-based snacks, extrusion cooking, food waste valorization, seafood by-products, sustainable food processing

## Abstract

Crispy fried shrimp legs are an underutilized by‐product generated during crispy shrimp head processing, accounting for approximately 50%–60% of the initial frozen shrimp weight. Rich in protein and calcium, this material has considerable potential for value‐added food applications; however, its use in extruded snack products has not previously been investigated. This study evaluated the feasibility of utilizing pretreated crispy fried shrimp leg powder as a functional ingredient in corn‐based extruded snacks. Shrimp leg powder was incorporated at two levels (15% and 20%), and the effects of screw speed (350–450 rpm) and final barrel temperature (110°C–130°C) on the physicochemical, textural, nutritional, microbiological, and sensory properties of the products were investigated. Greater shrimp leg powder incorporation generally reduced expansion and increased hardness because of interactions between proteins, minerals, chitin, and the starch matrix during extrusion. The optimal processing condition, determined using a multicriteria decision approach, was obtained with 15% shrimp leg powder, a screw speed of 400 rpm, and a final barrel temperature of 120°C. The optimized products exhibited low moisture content (2.84%–2.93% db) and water activity (0.150–0.157), high protein (10.33%–10.44% db) and calcium contents (835.60–857.50 mg/100 g), and complied with Thai microbiological standards for sealed packaged foods. Consumer sensory evaluation showed good overall acceptability, with respective overall liking scores of 7.62 and 7.68 for the original‐ and paprika‐flavored products. This study demonstrates, for the first time, that crispy fried shrimp leg by‐products can be successfully valorized into calcium‐rich extruded snacks, providing a sustainable strategy for seafood by‐product utilization and functional snack development.

## 1. Introduction

The global seafood processing industry generates large quantities of by‐products, including heads, shells, tails, and legs, which may represent approximately 40%–60% of the total raw material depending on species and processing method [1]. These materials are often discarded or used for low‐value applications such as animal feed or fertilizer, resulting in inefficient resource utilization and environmental concerns. However, seafood by‐products contain valuable nutrients and bioactive compounds, including proteins, minerals, chitin, and carotenoids, and hence offer significant potential for value‐added food applications [2]. Consequently, the valorization of seafood processing residues has attracted increasing attention as a sustainable strategy by which to reduce waste and improve resource efficiency.

Shrimp is one of the most widely consumed seafood products worldwide, and its processing generates waste materials in substantial quantities. At the same time, shrimp by‐products contain proteins and minerals at considerable levels, particularly calcium [1, 3], due to shrimp having mineralized exoskeletal structures composed mainly of chitin and calcium carbonate. Among these by‐products, shrimp legs remain relatively underutilized. In certain shrimp‐processing operations, such as crispy shrimp head production, fried shrimp legs may account for approximately 50%–60% of the initial frozen shrimp weight. Converting shrimp legs into food ingredients could therefore provide an effective approach for waste reduction and sustainable food production.

Extrusion cooking is widely used in the food industry to produce expanded snack products with desirable characteristics such as low density and crispy texture. Key processing parameters, including barrel temperature, screw speed, and feed moisture content, are critical in determining the structural, textural, and sensory quality of the final product [4]. In addition to cereal‐based ingredients, extrusion technology allows incorporation of alternative materials, including food‐processing by‐products [5]. Recent studies have explored the incorporation of protein‐ and fiber‐rich ingredients into extruded snacks to enhance their nutritional value [6–12]. Seafood‐derived ingredients, including fish powder and shrimp‐derived materials, have been incorporated into corn‐ and rice‐based extruded snacks with favorable effects on protein content and sensory acceptability [13]. However, these additions significantly alter the structural, textural, and sensory characteristics of the final products. In particular, higher protein and fiber contents have been reported to reduce expansion ratio, increase bulk density and hardness, and negatively affect crispiness and overall sensory acceptability due to interference with starch gelatinization and bubble formation during extrusion [4, 14]. The degree of these effects is modulated by extrusion process parameters, which govern starch gelatinization, protein denaturation, and the thermomechanical energy input during processing [4, 15]. Indeed, it has been demonstrated that optimizing process parameters is essential to balancing the nutritional benefits of enriched ingredients with the desirable physical and sensory qualities expected by consumers [15, 16].

Despite the overall growing interest in utilizing seafood by‐products in food products, only limited research has focused specifically on the application of shrimp leg by‐products in extruded snack formulations. Previous studies have mainly focused on shrimp meat powder, shrimp protein isolates, shrimp shell powder, or enzymatically hydrolyzed shrimp proteins as functional ingredients. Crispy fried shrimp legs represent a unique processing by‐product generated during crispy shrimp head production; compared with conventional shrimp by‐products, shrimp legs possess lower moisture content, enhanced roasted flavor, and partially modified protein–chitin structures resulting from the frying process, together with naturally high levels of protein and calcium‐rich exoskeletal minerals. These characteristics are expected to influence functionality during extrusion differently from untreated shrimp‐derived ingredients. To the best of our knowledge, this is the first study to investigate the use of crispy fried shrimp leg powder as a functional ingredient in corn‐based extruded snacks while systematically evaluating the interactive effects of ingredient level and extrusion processing conditions on product quality.

In precise terms, this study is aimed at developing calcium‐rich corn‐based extruded snacks by valorizing crispy fried shrimp leg powder, an underutilized by‐product generated during crispy shrimp‐head processing. The effects of shrimp leg powder incorporation level together with extrusion processing conditions (barrel temperature and screw speed) on the physicochemical, textural, nutritional, microbiological, and sensory properties of the resulting snacks were systematically evaluated to determine the feasibility of producing nutritionally enhanced extruded snacks while promoting the sustainable utilization of shrimp processing residues.

## 2. Materials and Methods

### 2.1. Raw Materials

The primary raw materials used in this study were corn grits, crispy fried shrimp legs, white granulated sugar, seasoning powder, white pepper powder, and paprika powder. Commercial corn grits were obtained from Yok Intertrade Chiang Mai Co. Ltd. (Chiang Mai, Thailand). Crispy fried shrimp legs were supplied by Prikhom Interfood Co. Ltd. (Nakhon Sawan, Thailand) and used as a partial substitute for corn grits in the snack formulation. These shrimp legs are generated as a by‐product during the centrifugation step used for oil removal in the production of crispy fried shrimp head products. During centrifugation, shrimp legs detach from the heads and are collected separately. The recovered legs are incidentally coated with residual batter and seasoning originating from the crispy shrimp head production process. To ensure sufficient material for the experiments, shrimp legs were delivered in five batches (15 kg per batch). The samples were packaged in laminated aluminum foil bags, placed in cardboard containers, and transported to the Department of Agro‐Industry, Faculty of Agriculture, Natural Resources and Environment, Naresuan University (Phitsanulok, Thailand). Upon arrival, the samples were stored at room temperature (25^°^C ± 5^°^C) prior to further analysis. Commercial white granulated sugar, seasoning powder, white pepper powder, and paprika powder were purchased from a local retail supplier.

### 2.2. Preparation of Shrimp Leg Powder

The crispy fried shrimp legs obtained as a by‐product of centrifugation carried residual batter and seasoning coating originating from the shrimp head production process. Since this coating was incidental rather than intrinsic to the shrimp leg material itself, a washing pretreatment was applied prior to powder production in the interest of obtaining a more representative and compositionally consistent shrimp leg ingredient. Batter coatings typically possess high water absorption and moisture retention capacity, and therefore may interfere with heat and pressure transfer during extrusion, consequently reducing product expansion and crispness. For the pretreatment, shrimp legs were washed with distilled water at a ratio of 1:5 (w/v) and stirred for 30 min using a propeller‐type mixer. The suspension was then filtered through a 20‐mesh sieve (860 *μ*m) and rinsed three times with distilled water to remove excess coating materials. Next, the cleaned samples were dried in a hot‐air oven at 70°C for 3.5 h, cooled to room temperature (moisture content ≈2.09% db), and ground using a laboratory grinder for approximately 10 s to obtain a powder with a particle size of approximately 40 mesh (≈400 *μ*m). The powder was finally dried at 70°C for 15 min to reduce residual moisture (moisture content ≈2.84% db), cooled, and packaged in aluminum foil bags (500 g per bag) under nitrogen flushing. Samples were stored at room temperature (25^°^C ± 5^°^C). The overall yield of the pretreatment process was approximately 48%–50%.

### 2.3. Physicochemical Characterization of Raw Materials

Crispy fried shrimp legs, shrimp leg powder, and corn grits were analyzed for selected physicochemical properties. Color was measured using a colorimeter (CR‐100, Minolta Co. Ltd., Japan) and expressed in CIE L^*^ (lightness), a^*^ (red–green), and b^*^ (yellow–blue) values. Proximate composition, including moisture, crude protein, crude fat, crude fiber, ash, and carbohydrate contents, was determined according to standard methods of AOAC International (2000). Water activity (a_w_) was measured using a water activity analyzer (Lab Swift‐aw, Novasina, Switzerland). Calcium content was determined after wet digestion (Modified AOAC 935.13) using atomic absorption spectrophotometry (ContrAA 800 D, Analytik Jena, Germany).

### 2.4. Formulation and Extrusion Processing of the Extruded Snack Products

The base formulation for crispy fried shrimp leg snacks consisted of 89.06% corn grits, 6.25% sugar, 3.13% seasoning powder, and 1.56% white pepper powder (w/w). Prior to extrusion, all dry ingredients were weighed and hand‐mixed for approximately 5 min. The feed moisture content of the formulation was adjusted to 8%–9% db and maintained constant throughout all extrusion experiments. No additional conditioning step was applied prior to feeding the mixture into the extruder.

Extrusion processing was performed using a twin‐screw extruder (CT, CTE‐D50L32, China) with a die diameter of 4 mm. The barrel consisted of seven heating zones. Corn grits were partially substituted with shrimp leg powder (moisture content 2.84% db; particle size ≈400 *μ*m) at two substitution levels (15% and 20% of the total formulation). Two extrusion parameters were investigated: screw speed (350, 400, and 450 rpm) and the temperature of the final barrel zone (110, 120°C, and 130°C). These parameters were selected as the independent variables because they are among the primary processing factors governing thermomechanical energy input, starch gelatinization, protein denaturation, expansion, and the textural properties of extruded products. The ranges utilized were established through preliminary trials to ensure stable extrusion of the formulation. Feed moisture content (8%–9% db), feed rate (4.20 kg/h), and the temperatures of the remaining barrel zones (40°C, 55°C, 80°C, 90°C, 100°C, and 100°C) were maintained constant throughout the experiments to isolate the effects of the selected processing variables.

Following extrusion, the products were dried in a hot‐air oven at 70°C for 15 min to reduce residual moisture from 8.16% to 1.7%–3.0% db. The drying temperature and time were determined based on preliminary trials to achieve a final moisture content suitable for maintaining crisp texture and shelf stability. The final dried samples were packaged in aluminum foil bags (100 g per bag), flushed with nitrogen gas to minimize oxidative deterioration, and stored at room temperature (25^°^C ± 5^°^C) until further analysis.

### 2.5. Analysis of Physical, Textural, and Functional Properties of the Extruded Snack Products

The physical, textural, and functional properties of the extruded snacks were evaluated in terms of appearance, color, texture, expansion ratio, bulk density, water absorption index (WAI), and water solubility index (WSI). Color was measured using a colorimeter (CR‐100, Minolta Co. Ltd., Japan) and expressed as CIE L^*^ (lightness), a^*^ (red–green), and b^*^ (yellow–blue) values. Texture properties, including hardness, crispness, adhesiveness, fracturability, and chewiness, were determined using a texture analyzer (TA.HDplus, Stable Micro Systems, United Kingdom) equipped with a 36‐mm cylindrical aluminum probe (P/36R). The instrument was operated under the following conditions: pretest speed 2 mm/s, test speed 1 mm/s, posttest speed 10 mm/s and compression distance 5 mm. Five replicate samples were analyzed for each treatment.

Expansion ratio, bulk density, WAI, and WSI were determined according to Singh et al. [17] with some modifications. The diameters of 10 randomly selected extrudates from each treatment were measured using a digital caliper (MW110‐15DBL, Moore & Wright, United Kingdom), and the average value was used to calculate the expansion ratio as the ratio of the extrudate diameter to the die diameter.

Bulk density was determined from the mass of 3‐cm‐long extrudate pieces according to the following equation:

Bulk density gcm3=4Mπd2L,

where *M* is mass (g), *d* is diameter (cm), and *L* is length (cm). The experiment was done in triplicate.

Water absorption was determined by dispersing 0.5 g of ground extrudate (passed through a 400‐*μ*m sieve) in 10 mL of distilled water. The suspension was stirred and allowed to stand for 30 min, with intermittent shaking every 5 min, then centrifuged at 10,000 rpm for 15 min using a refrigerated centrifuge (K240 R, Centurion Scientific, United Kingdom). Afterwards, the supernatant was carefully decanted and the sediment weighed. The WAI was calculated as follows:

WAI gg=weight of hydrated sedimentdry weight of sample.

The WSI was determined using the same suspension prepared for WAI analysis. After centrifugation, the supernatant was transferred into a preweighed aluminum dish and dried at 105°C until a constant weight was obtained. WSI was then calculated using the following equation:

WSI %=weight of dry solids in supernatant×100dry weight of extrudate.

### 2.6. Comparison of the Relationships Among Key Quality Attributes

Principal component analysis (PCA) was employed for comparison of the relationships among all key quality attributes, including moisture content, water activity, color, expansion ratio, density, WAI, WSI, and texture properties.

The optimal extrusion condition for producing crispy fried shrimp leg snacks was selected using a multicriteria decision approach based on the desirable expansion ratio, low bulk density, and low hardness texture. These parameters were prioritized as they are well‐established physical indicators of crispness and structural quality in extruded snack products and have been shown to correlate with sensory perception of crispiness and overall acceptability.

### 2.7. Preparation of Flavored Snack Products

After identifying the optimal extrusion condition, crispy fried shrimp leg snacks were produced in two flavor variants: original and paprika. The products were dried in a hot‐air oven at 70°C for 15 min and subsequently packaged in aluminum foil bags (100 g per bag) under nitrogen flushing. The packaged samples were stored at room temperature (25^°^C ± 5^°^C) until further analysis. The packaging and storage conditions were designed to comply with the requirements for hermetically sealed foods according to Thai Ministry of Public Health regulations concerning foods in sealed containers and to microbiological standards for food safety.

### 2.8. Chemical Analysis

Chemical properties of the snack samples were determined as follows. Moisture content, crude protein, crude lipid, and peroxide value were determined according to the methods of AOAC International [18]. Water activity was measured using a water activity analyzer (Novasina, Lab Swift‐aw, Switzerland). Calcium content was determined after wet digestion (modified AOAC 935.13) using atomic absorption spectrophotometry (ContrAA 800 D, Analytik Jena, Germany). Briefly, samples were prepared by dissolving in distilled water and acidifying with concentrated HCl. All standards and samples were prepared in 1% HCl and supplemented with H_2_SO_4_ (1.88 M) and 0.2% lanthanum chloride solution to suppress interferences. Calcium absorbance was measured at a wavelength of 422.7 nm using a N_2_O/acetylene flame with an ionization buffer of KCl/CsCl (0.1%) to minimize ionization interference.

### 2.9. Microbiological Analysis

Microbiological quality was evaluated to ensure compliance with food safety standards. Total plate count, yeast and mold, and coliform bacteria were determined according to AOAC International methods [19]. The presence of *Salmonella* spp. was determined according to ISO 6579 [20]. *Staphylococcus aureus*, *Bacillus cereus*, and *Clostridium perfringens* were analyzed according to methods described in the U.S. Food and Drug Administration Bacteriological Analytical Manual.

### 2.10. Sensory Evaluation

Sensory evaluation was conducted in accordance with the ethical guidelines of the Institutional Review Board, Naresuan University, Phitsanulok, Thailand and approved under ethical approval number IRB No. P1‐0167/2567. All procedures were performed in compliance with relevant institutional and national guidelines and regulations governing human subject research. Prior to participation, all panelists were fully informed of the study objectives and procedures, and written informed consent was obtained. Participation was entirely voluntary, and panelists were free to withdraw at any time without consequence. Inclusion criteria required panelists to be aged 18–59 years, have no known allergy to seafood (shrimp) or corn, be able to communicate in Thai, and demonstrate the ability to perceive basic tastes including sweet, salty, bitter, sour, and umami.

A 9‐point hedonic scale, range 1 (dislike extremely) to 9 (like extremely), was used by 50 untrained panelists to evaluate extruded snacks in original and paprika flavors for color, size, shape, odor, crispiness, hardness, sweetness, saltiness, and overall acceptability. Although the panelists were untrained, they were selected to represent the target consumer group for the developed snack products. Prior to evaluation, a brief orientation was provided to ensure that panelists understood the hedonic scale and the sensory attributes being assessed. The 9‐point hedonic scale was chosen as it measures overall personal preference and degree of liking rather than requiring technical discrimination ability, making it appropriate for use with consumer panels. Attributes such as crispiness, hardness, sweetness, and saltiness were evaluated based on individual perception, which reflects real‐world consumer experience and acceptance.

Prior to evaluation, samples were prepared and served in aluminum foil bags, with three pieces per serving, labeled with random three‐digit codes indicating the sample to be evaluated. One bottle of drinking water was provided for palate cleansing between samples. The evaluation was conducted at room temperature under normal lighting conditions.

### 2.11. Nutritional Analysis

The nutritional composition of the original and paprika flavored snack samples was determined by an accredited external laboratory, Central Laboratory (Thailand) Co. Ltd., Chiang Mai, Thailand.

### 2.12. Statistical Analysis

All experiments were conducted in triplicate. Physical and functional properties of the extruded snacks were performed in technical triplicate, whereas texture properties were analyzed using five replicate samples, all using aliquots taken from the same batch. Results are expressed as the mean ± standard deviation of the technical replicates. Data were analyzed using one‐way analysis of variance to determine significant differences among treatments. Mean comparisons were performed using Duncan′s multiple range test at a significance level of *p* < 0.05. Statistical analyses were carried out using IBM SPSS Statistics (Version 26, IBM Corp., Armonk, New York, United States).

## 3. Results and Discussion

### 3.1. Appearance and Color Characteristics of Raw Materials

The appearance of the main raw materials is illustrated in Figure 1. Crispy fried shrimp legs exhibited a light orange color that became darker after washing and grinding, whereas corn grits displayed a deep yellow color. These color differences are mainly associated with the natural carotenoid pigments present in each material: shrimp shells contain carotenoid pigments that contribute to orange–reddish hues, particularly astaxanthin [21, 22], whereas the color of corn grits is attributed to yellow carotenoids such as lutein and zeaxanthin [23, 24]. Carotenoids may also influence color development during thermal processing.

**Figure 1:**
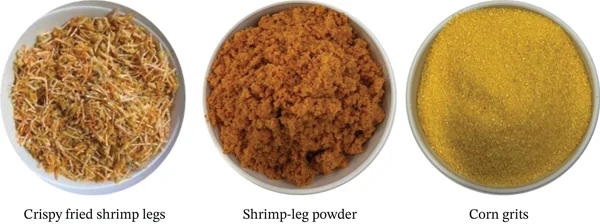
Appearance of crispy fried shrimp legs, shrimp‐leg powder, and corn grits.

The color characteristics of the raw materials are expected to influence the visual appearance of the extruded products. Accordingly, the final snacks were anticipated to exhibit a yellowish color with slight orange tones depending on the proportion of shrimp leg powder incorporated into the formulation. This expectation is consistent with the instrumental color measurements presented in Table 1, in that corn grits showed significantly higher L^*^ (lightness) and b^*^ (yellowness) values but lower a^*^ (redness) values compared with crispy fried shrimp legs and shrimp leg powder.

**Table 1:** Physicochemical properties of crispy fried shrimp legs, shrimp leg powder, and corn grits.

Sample	Color			Moisture content (% db)	Fat content (% db)	Protein content (% db)	Fiber content (% db)	Ash content (% db)	Carbohydrate content (% db)	Water activity	Calcium content (mg/100 g)
	L^*^	a^*^	b^*^								
Crispy fried shrimp legs	41.60 ± 0.02^c^	21.86 ± 0.07^a^	30.15 ± 0.12^c^	2.10 ± 0.01^c^	34.85 ± 0.83^a^	27.61 ± 0.08^a^	16.23 ± 0.67^b^	14.18 ± 0.05^a^	5.62 ± 1.52^b^	0.23 ± 0.01^c^	2855.00 ± 65.00^b^
Shrimp leg powder	45.60 ± 0.20^b^	18.59 ± 0.14^b^	31.06 ± 0.42^b^	2.84 ± 0.03^b^	34.59 ± 0.22^a^	27.04 ± 0.03^b^	22.54 ± 0.05^a^	11.18 ± 0.07^b^	1.81 ± 0.19^c^	0.41 ± 0.02^b^	5244.30 ± 63.00^a^
Corn grits	70.72 ± 0.09^a^	14.15 ± 0.13^c^	48.74 ± 0.25^a^	12.22 ± 0.05^a^	1.66 ± 0.02^b^	6.89 ± 0.00^c^	0.60 ± 0.02^c^	0.35 ± 0.00^c^	78.28 ± 0.04^a^	0.64 ± 0.00^a^	NA

*Note:* Values with different lowercase letters (a–c) within the same column indicate significant differences (*p* < 0.05). NA: not analyzed.

### 3.2. Chemical Composition of Raw Materials

The chemical compositions of crispy fried shrimp legs, shrimp leg powder, and corn grits are presented in Table 1. Shrimp‐derived materials contained higher levels of fat (34.59%–34.85% db), protein (27.04%–27.61% db), and calcium (2855.00–5244.30 mg/100 g), whereas corn grits were predominantly composed of carbohydrates (78.28% db) due to their starch‐rich composition. The two shrimp‐derived materials also exhibited significant differences in components, except fat content, with shrimp leg powder having significantly higher moisture, fiber, water activity, and calcium content than untreated crispy fried shrimp legs. These differences may be attributed to the washing process, which removed residual batter and coating materials attached to the shrimp legs; this reduced the overall carbohydrate fraction while increasing the relative proportion of shrimp shell constituents such as chitin and minerals, thereby resulting in higher fiber and calcium contents. Such compositional changes may influence extrusion performance as well as the nutritional quality of the final product. It should be noted, however, that the washing procedure was not fully standardized with regard to reproducibility across batches, and minor compositional variation between batches may occur depending on the degree of coating on the incoming raw material. Future studies should consider standardizing the pretreatment protocol to improve batch‐to‐batch consistency. Notably, the shrimp leg powder obtained in this study contained more calcium than reported for milk [25] and small whole fish [26], at approximately 19.66 and 2.30 times greater, respectively. These findings suggest that shrimp leg powder may serve as a potential ingredient for the development of calcium‐enriched food products. Nevertheless, further studies are needed to evaluate the bioavailability of calcium from shrimp leg powder.

Corn grits exhibited the highest moisture content (12.22% db), which is below the maximum moisture limit of 15% set for maize grits by the Codex Alimentarius [27]. The a_w_ of the raw materials ranged from 0.23 to 0.64. Moisture content and a_w_ are critical parameters influencing extrusion behavior and final product characteristics; in particular, appropriate moisture levels promote stable extrusion flow, starch gelatinization, and product expansion, whereas excessively high or low moisture may adversely affect expansion and texture [28, 29]. The relatively low a_w_ and suitable moisture content observed in this study indicate that the raw materials are appropriate for extrusion processing and can be safely stored prior to further processing.

It should be noted that the washing pretreatment, although technically necessary to remove the incidental batter and oil coating and ensure extrusion efficacy, resulted in an overall yield of approximately 48%–50% of the incoming shrimp leg material. The mass loss predominantly reflects removal of batter, seasoning, and residual frying oil rather than loss of the shrimp leg structural components, as evidenced by the high protein and calcium contents retained in the powder. However, the resulting wash water was not characterized in this study and was discarded, representing an unquantified secondary waste stream. From a full circular‐economy perspective, future studies should investigate the composition of this wash water and explore its potential valorization to further enhance the overall resource efficiency and sustainability of the process.

### 3.3. Optimal Level of Shrimp Leg Powder, Screw Speed, and Barrel Temperature for Extrusion Processing

#### 3.3.1. Appearance of Extruded Snack Products

The appearance of the 18 extruded snack samples is depicted in Figure 2. Differences in expansion and color were visually evident among the products, which may be attributed to variation in shrimp leg powder content, screw speed, and barrel temperature during extrusion. In general, higher barrel temperatures produced darker extrudates. This color development is likely associated with thermal reactions occurring during extrusion, particularly Maillard reactions between reducing sugars and amino acids and caramelization of carbohydrates at elevated temperatures. These reactions generate brown pigments, resulting in products becoming darker as processing temperature increases [28, 29]. In addition, the incorporation of protein‐rich shrimp materials may further promote Maillard browning, contributing to the observed color differences among samples.

**Figure 2:**
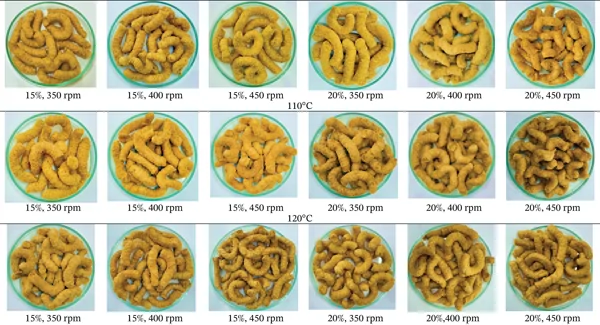
Physical appearance of extruded snack products made from crispy fried shrimp legs.

#### 3.3.2. Moisture Content

The moisture content of the extruded snacks ranged from 1.08% to 3.28% (db) (Table 2). Increasing shrimp leg powder content from 15% to 20%, screw speed from 350 to 450 rpm, and final barrel temperature from 110°C to 130°C generally reduced product moisture. This reduction may be attributed to the higher protein, fiber, and mineral content of shrimp leg powder, which decreases the water‐holding capacity of the starch matrix. In addition, higher screw speed increases mechanical shear, promoting starch gelatinization and moisture evaporation during extrusion, and elevated barrel temperature enhances heat transfer and internal pressure, leading to rapid flash evaporation of water as the melt exits the die [28, 29]. Consequently, the snacks exhibited very low moisture levels, which is desirable for extruded products. Moisture contents below 10% are associated with improved crispness, sensory acceptability, and shelf stability due to reduced microbial growth and slower quality deterioration [30, 31].

**Table 2:** Physicochemical properties of extruded snack products made from crispy fried shrimp legs.

Sample		Moisture content (% db)	Water activity	Color			Expansion ratio	Bulk density (g/cm^3^)	Water absorption index (%)	Water solubility index (%)
				L^*^	a^*^	b^*^				
1	15%, 350 RPM, 110°C	2.70 ± 0.28^bc^	0.137 ± 0.04^c^	43.88 ± 0.54^bcd^	5.94 ± 0.33^efg^	21.63 ± 0.34^cde^	2.39 ± 0.12^abc^	0.16 ± 0.01^bc^	2.91 ± 0.17^de^	27.84 ± 0.67^gh^
2	15%, 400 RPM, 110 °C	2.53 ± 0.14^bcd^	0.171 ± 0.03^bc^	42.55 ± 1.27^de^	6.13 ± 0.78^ef^	21.89 ± 1.17^bcd^	2.19 ± 0.20^de^	0.16 ± 0.02^bc^	3.04 ± 0.07^cde^	28.88 ± 0.86^efg^
3	15%, 450 RPM, 110 °C	1.08 ± 0.96^g^	0.133 ± 0.01^c^	33.55 ± 1.46^k^	5.27 ± 0.07^h^	16.98 ± 0.57^j^	2.27 ± 0.16 cd	0.14 ± 0.02^c^	3.34 ± 0.07^abc^	24.70 ± 0.59^j^
4	20%, 350 RPM, 110 °C	2.55 ± 0.13^bcd^	0.158 ± 0.03^bc^	39.48 ± 0.87^gh^	5.39 ± 0.29^gh^	18.27 ± 0.57^i^	2.12 ± 0.07^ef^	0.20 ± 0.08^b^	2.76 ± 0.43^e^	26.06 ± 1.30^ij^
5	20%, 400 RPM, 110 °C	2.44 ± 0.12^cd^	0.146 ± 0.04^c^	41.92 ± 1.78^ef^	6.81 ± 0.53^bcd^	21.24 ± 0.50^def^	2.04 ± 0.09^f^	0.24 ± 0.09^a^	3.51 ± 0.25^a^	26.44 ± 0.66^hi^
6	20%, 450 RPM, 110 °C	2.34 ± 0.12^cde^	0.144 ± 0.04^c^	44.20 ± 0.40^abc^	7.38 ± 0.04^ab^	22.45 ± 1.12^bc^	2.20 ± 0.03^de^	0.19 ± 0.04^bc^	3.36 ± 0.09^abc^	32.62 ± 1.04^a^
7	15%, 350 RPM, 120°C	3.28 ± 0.95^a^	0.131 ± 0.02^c^	45.55 ± 1.43^a^	6.90 ± 0.28^bcd^	24.08 ± 0.99^a^	2.35 ± 0.13^bc^	0.15 ± 0.02^bc^	3.34 ± 0.25^abc^	22.88 ± 1.54^k^
8	15%, 400 RPM, 120°C	3.00 ± 0.45^ab^	0.156 ± 0.05^bc^	35.99 ± 0.96^j^	6.11 ± 0.75^ef^	19.20 ± 1.20^hi^	2.51 ± 0.04^a^	0.15 ± 0.01^bc^	3.35 ± 0.08^abc^	24.81 ± 1.44^ij^
9	15%, 450 RPM, 120°C	2.20 ± 0.11^cde^	0.229 ± 0.07^a^	43.60 ± 0.66^gh^	5.79 ± 0.46^fgh^	19.70 ± 0.35^gh^	2.43 ± 0.06^ab^	0.14 ± 0.02^c^	3.07 ± 0.33^cd^	32.29 ± 2.58^ab^
10	20%, 350 RPM, 120°C	2.47 ± 0.10^cd^	0.156 ± 0.02^bc^	41.60 ± 0.66^ef^	5.42 ± 0.21^gh^	19.95 ± 1.86^gh^	2.27 ± 0.02^cd^	0.18 ± 0.02^bc^	3.36 ± 0.14^abc^	28.43 ± 0.96^fg^
11	20%, 400 RPM, 120°C	2.32 ± 0.22^cde^	0.147 ± 0.02^c^	39.04 ± 0.57^hi^	6.37 ± 0.60^def^	20.36 ± 1.26^fgh^	2.16 ± 0.11^def^	0.16 ± 0.03^bc^	3.52 ± 0.11^a^	30.33 ± 0.67^cde^
12	20%, 450 RPM, 120°C	2.10 ± 0.13^de^	0.157 ± 0.02^bc^	42.95 ± 1.45^cde^	7.36 ± 0.15^abc^	20.51 ± 1.65^efg^	2.27 ± 0.09^cd^	0.16 ± 0.02^bc^	3.44 ± 0.88^ab^	32.65 ± 0.96^a^
13	15%, 350 RPM, 130°C	2.20 ± 0.11^cde^	0.141 ± 0.01^c^	37.75 ± 1.19^i^	6.92 ± 0.29^bcd^	19.31 ± 1.71^ghi^	2.06 ± 0.09^ef^	0.16 ± 0.03^bc^	3.50 ± 0.12^a^	29.17 ± 1.19^defg^
14	15%, 400 RPM, 130°C	2.11 ± 0.24^de^	0.164 ± 0.02^bc^	45.09 ± 1.75^ab^	6.12 ± 0.25^ef^	21.61 ± 0.28^cde^	1.83 ± 0.09^g^	0.16 ± 0.01^bc^	3.29 ± 0.35^abc^	31.11 ± 1.07^abc^
15	15%, 450 RPM, 130°C	1.52 ± 0.16^fg^	0.136 ± 0.01^c^	40.91 ± 0.95^fg^	6.43 ± 0.59^de^	20.11 ± 0.39^fgh^	1.76 ± 0.12^g^	0.18 ± 0.06^bc^	3.06 ± 0.12^cde^	32.20 ± 1.14^ab^
16	20%, 350 RPM, 130°C	1.85 ± 0.42^ef^	0.133 ± 0.01^c^	39.15 ± 1.87^hi^	6.77 ± 0.19^cd^	19.46 ± 0.36^gh^	2.06 ± 0.12^ef^	0.19 ± 0.02^bc^	2.88 ± 0.17^de^	25.61 ± 1.60^ij^
17	20%, 400 RPM, 130°C	1.46 ± 0.19^fg^	0.144 ± 0.01^c^	39.12 ± 0.82^hi^	7.11 ± 0.27^abc^	19.95 ± 0.41^gh^	2.07 ± 0.11^ef^	0.17 ± 0.02^bc^	3.29 ± 0.09^abc^	30.10 ± 1.58^cdef^
18	20%, 450 RPM, 130°C	1.57 ± 0.29^fg^	0.144 ± 0.02^c^	45.11 ± 1.04^ab^	7.68 ± 0.46^a^	22.97 ± 0.63^b^	2.35 ± 0.02^bc^	0.15 ± 0.01^bc^	3.12 ± 0.07^bcd^	30.67 ± 1.11^bcd^

*Note:* Values with different lowercase letters (a–k) within the same column indicate significant differences (*p* < 0.05).

#### 3.3.3. Water Activity

Water activity is an important parameter affecting the microbial stability of snack products. In this study, a_w_ values ranged from 0.131 to 0.229 (Table 2). Increasing shrimp leg content from 15% to 20% did not in general significantly affect a_w_; however, specifically under the conditions of 450 rpm screw speed and 120°C barrel temperature, increasing shrimp leg content reduced a_w_ from 0.229 to 0.157.

Screw speed (350–450 rpm) and final barrel temperature (110°C–130°C) generally had no significant effect on a_w_. Under all 18 processing conditions, a_w_ values remained below 0.60, indicating low potential for the growth of most pathogenic microorganisms during storage [32]. However, microbiological quality was evaluated separately through microbiological analyses (Section 3.4.2), and all tested parameters complied with the limits specified by the Thai Ministry of Public Health Notifications No. 355 (2013) and No. 416 (2020).

#### 3.3.4. Color of Extruded Snack Products

The color characteristics of the extruded snacks are presented in Table 2. Lightness (L^*^) values ranged from 33.55 to 45.11, indicating noticeable differences in brightness among samples. Increasing shrimp leg powder content from 15% to 20% generally reduced L^*^ values, resulting in darker products (Figure 2); this may be attributed to the darker color of shrimp components, including proteins, chitin, and minerals, in comparison with corn‐based ingredients. In addition, thermal processing during extrusion can promote Maillard reactions between amino acids and reduce sugars, contributing to darker products [33]. This is consistent with the findings of Jeyakumari et al. [3], who reported a decrease in lightness (L^*^) with increasing shrimp protein hydrolysate and shrimp powder incorporation in rice‐corn–based extruded snacks, attributing the darker color to the presence of shrimp‐derived pigments and enhanced Maillard browning during extrusion. Similarly, Kumar et al. [6] observed that increasing paste shrimp (*Acetes* spp.) powder content resulted in darker extrudates, in association with greater carotenoid pigment content and the promotion of protein‐reducing sugar interactions at higher extrusion temperatures.

Increasing screw speed from 350 to 450 rpm tended to increase L^*^, possibly due to greater expansion and formation of air cells that enhance light reflection [34]. In contrast, increasing the final barrel temperature from 110 to 130°C decreased L^*^ on account of higher temperatures promoting browning reactions such as Maillard reactions and caramelization [35].

Redness (a^*^) values ranged from 5.27 to 7.68, indicating all samples to exhibit a reddish color. Increasing shrimp leg powder content increased a^*^ values, which can be attributed to the higher concentration of carotenoid pigments naturally present in shrimp legs, particularly astaxanthin [36]. Higher screw speed and barrel temperature also tended to increase a^*^, likely because of improved dispersion of carotenoid pigments and enhancement of nonenzymatic browning reactions during extrusion.

Yellowness (b^*^) values ranged from 16.98 to 24.08. Increasing shrimp leg powder content, screw speed, and barrel temperature generally decreased b^*^, which may be attributed to reduced contribution of yellow carotenoid pigments from corn and the formation of darker‐colored compounds through nonenzymatic browning reactions during extrusion [37].

#### 3.3.5. Expansion Ratio

The expansion ratio of the extruded snacks ranged from 2.04 to 2.51 (Table 2). Increasing shrimp leg powder content from 15% to 20% reduced expansion from 2.39 to 2.12; this decrease may be attributed to higher protein, mineral, and dietary fiber contents, which increase structural density and reduce steam retention during extrusion. Similar reductions in expansion have been reported for extruded cereal snacks enriched with dietary fiber [38], and for increasing fractions of shrimp‐derived ingredients in similar studies. Jeyakumari et al. [3] observed that higher levels of shrimp protein hydrolysate and shrimp powder in rice‐corn extrudates reduced expansion due to starch–protein interactions that limit bubble growth during extrusion. Kumar et al. [6] similarly reported that increasing paste shrimp powder content reduced the expansion ratio of extrudates, which they attributed to the higher protein content interfering with starch gelatinization and steam retention at the die exit. The present findings are therefore consistent with the broader trend observed in shrimp‐based extruded products.

Increasing the screw speed from 350 to 450 rpm generally had no significant effect on expansion. However, at 15% shrimp leg powder content and a barrel temperature of 130°C, the expansion ratio decreased from 2.06 to 1.76, which may be attributed to the shorter residence time at the higher screw speed resulting in less complete starch gelatinization and reduced bubble growth during extrusion.

Increasing the final barrel temperature from 110°C to 120°C enhanced the expansion ratio (2.04–2.51), which can be attributed to more extensive starch gelatinization and protein denaturation that in turn improved melt elasticity, steam retention, and expansion at the die exit [33, 39]. However, further increasing barrel temperature to 130°C reduced the expansion ratio, which effect may be associated with excessive starch degradation, reduced melt viscosity, and subsequent structural collapse or restricted bubble growth during expansion and cooling [40].

#### 3.3.6. Density

Consistent with the expansion ratio results, the density of the extruded snacks ranged from 0.14 to 0.24 g/cm^3^ (Table 2). Increasing shrimp leg powder content (15%–20%), screw speed (350–450 rpm), or final barrel temperature (110°C–130°C) did not significantly affect density, indicating that these processing conditions are insufficient to alter the melt structure of the products. Similar observations have been reported for starch‐based extruded snacks [39]. The density values obtained in this study are comparable with those reported for other extruded snacks (0.0989–0.229 g/cm^3^) and fall within the range generally accepted by consumers [41, 42].

The physical properties of extruded snacks, particularly expansion ratio and bulk density, are closely related to the internal structure formed during extrusion. Although microstructural analysis was not conducted in this study, the measured expansion ratio and density provide indirect indications of structural characteristics. A higher expansion ratio generally reflects a more porous structure with larger air cells, whereas higher density indicates a more compact structure with limited expansion [28, 29]. The presence of shrimp leg powder, which contains chitin and minerals, may interfere with starch gelatinization, disrupt the continuous starch matrix, and thereby restrict bubble growth during extrusion; that may in turn lead to reduced expansion and increased density. Similar effects have been reported in fiber‐enriched extruded products, where nonstarch components limit melt expansion [38]. Nevertheless, further studies using microstructural techniques, such as scanning electron microscopy, would be of benefit in better understanding the internal structure of the extrudates.

#### 3.3.7. WAI

The WAI of the extruded snacks ranged from 2.76 to 3.52 (Table 2). At 110°C and 120°C, increasing shrimp leg powder content from 15% to 20% tended to increase WAI, which may be attributed to enhanced starch gelatinization together with increased water retention by denatured proteins and starch–protein interactions formed during extrusion [33]. However, at 130°C, the WAI decreased, which may be associated with excessive starch dextrinization and protein aggregation that results in reduced capacity to bind water [43].

#### 3.3.8. WSI

The WSI indicates the number of soluble components released after processing and reflects the degradation of starch, proteins, and other macromolecules. Increasing shrimp leg powder content from 15% to 20% generally reduced WSI (Table 2). This may be attributed to the high levels of protein, chitin, and minerals in shrimp legs, which are largely insoluble in water and reduce the proportion of soluble starch derived from corn grits. Heat‐induced Maillard reactions may additionally promote cross‐linking between starch and proteins, forming larger molecules that are less soluble in water [44].

Meanwhile, increasing the screw speed from 350 to 450 rpm and the final barrel temperature from 110°C to 130°C increased the WSI. These processing conditions likely intensified mechanical shear and thermal degradation, resulting in depolymerization of starch and protein macromolecules, enhanced starch dextrinization, and the generation of low–molecular‐weight, water‐soluble compounds, thereby increasing the WSI [33].

Overall, the formulation and extrusion processing conditions played critical roles in determining the physicochemical and functional properties of the extruded snack products. Increasing the proportion of crispy fried shrimp legs influenced the structural composition of the product, resulting in changes in expansion behavior, texture, water absorption, and solubility. In addition, variations in screw speed and barrel temperature affected starch gelatinization, protein interactions, and molecular degradation during extrusion. These interactions collectively shaped the internal structure and quality characteristics of the final extruded snacks; as such, selecting appropriate formulation ratios and processing conditions is essential to obtaining desirable product quality and functionality.

#### 3.3.9. Texture of Extruded Snack Products

Texture is a key quality attribute of extruded snacks, affecting consumer acceptance. Texture profile analysis included hardness, fracturability, springiness, cohesiveness, gumminess, and chewiness, as shown in Table 3. Increasing the proportion of shrimp leg powder from 15% to 20% increased hardness from 3,603.64 to 5,027.11 g/force. This may be attributed to the higher protein, chitin, and mineral contents of shrimp leg powder, which reduces the proportion of corn grits and limits product expansion, resulting in a denser structure. These findings agree with those of Kumar et al. [6], who reported that greater shrimp powder incorporation significantly increased the hardness of extrudates, attributing this effect to the role of shrimp proteins in promoting protein network formation and cross‐linking of lysine and arginine residues during thermal processing, which resulted in a more compact and rigid structure. Increasing the screw speed from 350 to 450 rpm generally decreased hardness, which may be attributed to the greater mechanical shear promoting more extensive starch gelatinization and expansion, thereby producing a lighter and more porous cellular structure. Conversely, increasing the final barrel temperature from 110°C to 130°C increased hardness, which may be associated with enhanced starch cooking, moisture loss, and cell wall thickening that resulted in a denser and more robust mechanically stronger product structure [29].

**Table 3:** Texture of extruded snack products made from crispy fried shrimp legs.

Sample		Hardness (g/force)	Fracturability (g/force)	Springiness (g/force)	Cohesiveness (g/force)	Gumminess (g/force)	Chewiness (g/force)
1	15%, 350 RPM, 110°C	3603.64 ± 41.47^g^	3562.91 ± 65.95^fg^	0.15 ± 0.01^b^	0.03 ± 0.02^a^	124.28 ± 57.44^abcd^	19.10 ± 10.65^a^
2	15%, 400 RPM, 110 °C	2755.10 ± 42.54^kl^	2612.35 ± 120.69^k^	0.14 ± 0.03^b^	0.03 ± 0.01^a^	90.64 ± 40.10^cd^	13.52 ± 7.37^a^
3	15%, 450 RPM, 110 °C	2937.59 ± 81.43^jk^	2948.62 ± 190.44^ijk^	0.17 ± 0.04^ab^	0.04 ± 0.01^a^	104.59 ± 24.08^bcd^	18.81 ± 7.18^a^
4	20%, 350 RPM, 110 °C	5027.11 ± 53.76^b^	5010.21 ± 186.43^b^	0.11 ± 0.02^b^	0.04 ± 0.02^a^	164.71 ± 100.38^abc^	24.42 ± 29.21^a^
5	20%, 400 RPM, 110 °C	4747.67 ± 250.48^c^	4624.41 ± 412.98^c^	0.12 ± 0.02^b^	0.04 ± 0.01^a^	184.37 ± 51.00^ab^	22.29 ± 3.49^a^
6	20%, 450 RPM, 110 °C	3458.47 ± 96.82^gh^	3373.94 ± 91.56^gh^	0.11 ± 0.01^b^	0.02 ± 0.01^a^	78.36 ± 32.16^cd^	8.55 ± 3.74^a^
7	15%, 350 RPM, 120°C	2864.32 ± 169.75^kl^	2942.48 ± 226.66^ijk^	0.13 ± 0.01^b^	0.02 ± 0.01^a^	61.31 ± 23.91^d^	7.70 ± 2.84^a^
8	15%, 400 RPM, 120°C	2939.87 ± 67.64^jk^	3093.71 ± 84.22^hij^	0.16 ± 0.01^ab^	0.03 ± 0.01^a^	80.38 ± 23.05^cd^	13.19 ± 4.62^a^
9	15%, 450 RPM, 120°C	3300.25 ± 161.18^i^	3126.45 ± 227.13^hij^	0.15 ± 0.02^ab^	0.03 ± 0.01^a^	100.86 ± 28.94^bcd^	15.88 ± 5.97^a^
10	20%, 350 RPM, 120°C	2846.25 ± 53.73^kl^	2946.53 ± 208.34^ijk^	0.13 ± 0.05^b^	0.02 ± 0.01^a^	54.78 ± 32.28^d^	8.92 ± 8.88^a^
11	20%, 400 RPM, 120°C	4429.32 ± 166.07^d^	4482.70 ± 192.71^cd^	0.13 ± 0.04^b^	0.03 ± 0.01^a^	121.29 ± 38.67^abcd^	15.74 ± 4.95^a^
12	20%, 450 RPM, 120°C	2635.39 ± 152.62^l^	2752.90 ± 36.77^jk^	0.14 ± 0.01^b^	0.02 ± 0.01^a^	60.71 ± 26.45^d^	8.85 ± 4.42^a^
13	15%, 350 RPM, 130°C	4174.45 ± 46.90^e^	4163.88 ± 44.62^de^	0.13 ± 0.02^b^	0.03 ± 0.01^a^	115.21 ± 28.87^abcd^	14.93 ± 2.95^a^
14	15%, 400 RPM, 130°C	3850.68 ± 182.68^f^	3838.32 ± 126.81^ef^	0.14 ± 0.04^b^	0.03 ± 0.02^a^	124.63 ± 83.11^abcd^	20.23 ± 17.97^a^
15	15%, 450 RPM, 130°C	5663.07 ± 259.02^a^	5619.38 ± 263.41^a^	0.14 ± 0.01^b^	0.05 ± 0.01^a^	205.84 ± 48.33^a^	27.47 ± 5.86^a^
16	20%, 350 RPM, 130°C	3347.32 ± 170.66^hi^	3286.41 ± 212.67^ghi^	0.13 ± 0.03^b^	0.02 ± 0.01^a^	69.56 ± 25.41^d^	9.77 ± 4.95^a^
17	20%, 400 RPM, 130°C	4478.90 ± 132.09^d^	4512.37 ± 243.27^cd^	0.22 ± 0.12^a^	0.03 ± 0.01^a^	127.62 ± 32.62^abcd^	25.38 ± 8.06^a^
18	20%, 450 RPM, 130°C	4468.17 ± 221.46^d^	4518.05 ± 306.59^cd^	0.14 ± 0.03^b^	0.04 ± 0.02^a^	163.52 ± 67.48^abc^	22.45 ± 10.41^a^

*Note:* Values with different lowercase letters (a–l) within the same column indicate significant differences (*p* < 0.05).

Fracturability followed a similar trend to hardness, increasing with higher shrimp leg powder content. However, it was reduced with increasing screw speed at 20% shrimp leg powder content and 110°C, likely due to greater expansion and the formation of a more porous structure that fractures under lower force [45]. Higher barrel temperatures generally increased fracturability.

Springiness values were low, which is typical for expanded crispy snacks. Increasing shrimp leg powder content reduced springiness and cohesiveness due to the formation of a more rigid structure; higher screw speed slightly increased springiness through improved expansion; and increasing barrel temperature reduced springiness because rapid moisture loss produced a harder and less elastic structure. Cohesiveness and chewiness remained low and were not significantly affected by processing conditions. Gumminess increased with higher shrimp leg powder content and higher barrel temperature due to the formation of denser structures, whereas greater screw speed reduced gumminess because of the more porous structure of the extruded snacks.

Overall, the results indicate that both formulation and extrusion parameters are important in determining the texture of shrimp‐based extruded snacks; correspondingly, appropriate control of these factors is necessary to achieve desirable crispness and brittleness.

However, it should be noted that all 18 treatment combinations were statistically compared simultaneously using a one‐way ANOVA with Duncan′s multiple range test. The individual effects of shrimp leg powder level, screw speed, and barrel temperature discussed in the following sections were therefore interpreted descriptively by observing patterns in the Duncan′s groupings across treatment combinations sharing common factor levels, rather than through formal statistical partitioning of main effects or interactions. This represents a limitation of the current statistical analysis, and the observed trends should be interpreted with this in mind.

#### 3.3.10. Comparison of the Relationships Among Key Quality Attributes

The relationships among physicochemical, textural, and processing parameters involved in the production of extruded snack products from crispy fried shrimp legs were evaluated by PCA. The rotated component plot (Figure 3) illustrates the distribution of variables across the first three principal components.

**Figure 3:**
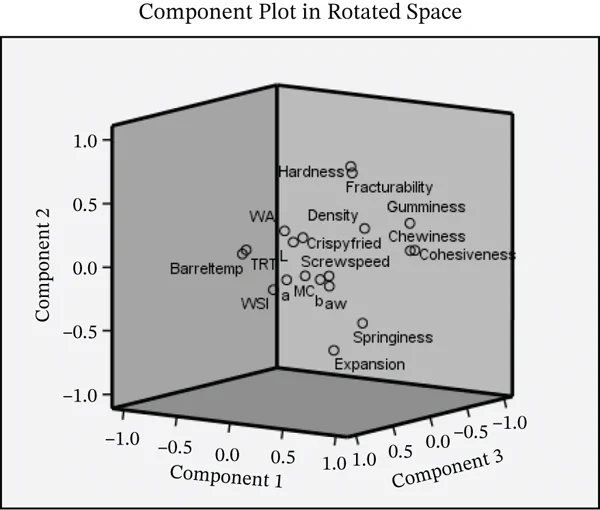
Principal components analysis of all parameters associated with production of extruded snack products from crispy fried shrimp legs.

Component 1 was mainly associated with moisture‐related properties, including moisture content, a_w_, and the WSI, indicating that these parameters are closely related and influenced by extrusion conditions. Processing variables such as screw speed and shrimp leg content were also located near this component, suggesting they contribute to changes in moisture behavior during extrusion.

Component 2 was primarily related to structural and mechanical properties, including hardness, fracturability, density, gumminess, and chewiness. The close grouping of these variables indicates strong relationships of product density with texture characteristics, reflecting the compactness of the extruded structure.

Component 3 was mainly associated with expansion and springiness. The negative relationship of expansion with density‐related texture parameters suggests that products with higher expansion tend to have a lighter, more porous structure and lower mechanical resistance.

In addition to clustering of variables, the PCA results also provide insight into the relationships among expansion behavior and textural properties of the extruded snacks. Samples showing higher expansion ratios tended to be located opposite hardness and density variables in the PCA plot. This indicates that greater expansion during extrusion produced a more porous internal structure, which resulted in lower density and reduced mechanical resistance during compression. Consequently, products with higher expansion generally exhibited lower hardness and gumminess, characteristics typical of crispy extruded snacks.

The distribution of samples in the PCA plot also suggests the incorporation of shrimp leg powder to influence the structural properties of the extruded products. Shrimp leg powder contains relatively high levels of protein, minerals, and chitin, which may interact with the starch matrix during extrusion; specifically, these components can limit bubble growth and reduce steam retention during the expansion process, leading to a denser product structure. As a result, samples with more shrimp leg powder tended to be associated with higher hardness and lower expansion values.

Overall, the PCA results confirm that the quality characteristics of the extruded snacks are mainly governed by the balance between starch expansion and the structural effects introduced by shrimp‐derived components. Proper control of formulation and processing conditions is therefore important to achieving a desirable combination of expansion, crispness, and mechanical stability in the final product.

#### 3.3.11. Selection of Optimal Processing Conditions

Optimal extrusion conditions were selected primarily based on the expansion ratio, a key indicator of the structural quality and crispness of extruded snack products. In light of the interaction effects of shrimp leg powder content, screw speed, and final barrel temperature, three processing conditions produced relatively high expansion ratios: (1) 15% shrimp leg powder, 350 rpm, and 110°C; (2) 15% shrimp leg powder, 400 rpm, and 120°C; and (3) 15% shrimp leg powder, 450 rpm, and 120°C. The density values of the snacks produced under these three conditions were not significantly different (Table 2).

Of these three conditions, 15% shrimp leg powder, 400 rpm screw speed, and 120°C final barrel temperature were identified as the optimal processing condition when other quality attributes were also considered (Tables 2 and 3). Specifically, this condition provided a high expansion ratio, low product density, and relatively lower hardness compared with the other conditions, indicating a lighter and crispier structure that is desirable for consumer acceptance.

### 3.4. Chemical, Microbiological, Sensory, and Nutritional Properties of Original‐ and Paprika‐Flavored Crispy Fried Shrimp Leg Snacks

#### 3.4.1. Chemical Properties

The chemical composition of extruded snacks made from crispy fried shrimp legs in original and paprika flavors is presented in Table 4. The moisture contents were 2.84 and 2.93% db, whereas the water activity ranged from 0.150 to 0.157. These low values are characteristic of extruded snack products and contribute to a crispy texture and enhanced microbiological stability [31].

**Table 4:** Chemical properties of original‐ and paprika‐flavored extruded snacks.

Samples	Moisture content (% db)	Water activity	Protein content (% db)	Fat content (% db)	Calcium content (mg/100 g)	Peroxide value (mEq/kg)
Original flavored	2.84 ± 0.27	0.150 ± 0.01	10.44 ± 0.01	2.15 ± 0.11	857.50 ± 0.01	0.00 ± 0.00
Paprika flavored	2.93 ± 0.29	0.157 ± 0.01	10.33 ± 0.01	2.38 ± 0.05	835.60 ± 0.01	0.00 ± 0.00

The protein contents were 10.44 and 10.33% db for the original and paprika flavors, respectively, whereas the fat contents were relatively low at 2.15 and 2.38% db. Respective calcium contents were 857.50 and 835.60 mg/100 g, reflecting the high calcium content of the shrimp leg powder incorporated into the formulation, which is rich in calcium carbonate complexes present in the shrimp exoskeleton. Compared with the calcium‐fortified freeze‐dried yogurt snacks reported by Wang et al. [46], the shrimp leg snacks have a calcium content approximately 1.13 times higher; this is above the lower end but substantially below the upper end of the range reported for extruded snacks supplemented with crab‐processing by‐products (510–5,240 mg/100 g), depending on the level of by‐product incorporation [47]. These differences are likely attributable to variations in the type, mineral composition, and incorporation level of the crustacean by‐products used; nevertheless, the relatively high calcium content confirms that shrimp leg powder is an effective natural source of dietary calcium, owing to the abundant mineralized chitin–calcium carbonate complexes present in the shrimp exoskeleton. It should also be noted that the fat content was substantially lower than that of conventional fried snacks (20%–40%) and within the typical range for extruded snacks (1%–5%) [29], indicating a nutritionally favorable snack product with desirable crispiness. The fat content of the final extruded snacks in this study was substantially lower than that of the raw shrimp leg powder (34.59% db). This reduction may be attributed to the dilution effect of the low‐fat corn grit base, constituting 85% of the formulation and fat losses during extrusion and postextrusion drying through fat migration and thermal oxidation under high‐temperature processing conditions. Finally, the elevated protein content is consistent with findings from Kumar et al. [6] and Jeyakumari et al. [3], both of whom reported improved protein content in extruded snacks upon incorporation of shrimp‐derived materials.

However, it should be noted that these chemical properties were analyzed from the same production batch, and all analyses were performed in triplicate. The reported values therefore reflect analytical repeatability within a single production batch rather than batch‐to‐batch variability.

#### 3.4.2. Microbiological Properties

The microbiological quality of the extruded snacks made from crispy fried shrimp legs in original and paprika flavors is summarized in Table 5. The total plate count of the paprika‐flavored snack was 6 × 10^1^ cfu/g, whereas yeasts and molds in the original‐flavored product were detected at 3 × 10^1^ cfu/g. *Salmonella* spp. and *S. aureus* were not detected in 25 g and 0.1 g of the samples, respectively, for both flavors. *B. cereus* and *C. perfringens* were detected, albeit at levels lower than 100 cfu/g, and coliform bacteria were less than 3 MPN/g.

**Table 5:** Microbiological properties of original‐ and paprika‐flavored extruded snacks.

Samples	Total plate count (cfu/g)	Yeasts and molds (cfu/g)	*Salmonella* spp. (cfu/25 g)	*Staphylococcus aureus* (cfu/g)	*Bacillus cereus* (cfu/g)	*Clostridium perfringens* (cfu/g)	Coliform bacteria (MPN/g)
Original flavored	^*^	3x10^1^	^**^	^*^	< 100	< 100	< 3
Paprika flavored	6x10^1^	^*^	^**^	^*^	< 100	< 100	< 3

*Note:* The asterisk “^*^” indicates not detected in a 0.1 g sample; “**” indicate not detected.

All microbiological parameters complied with the microbiological criteria specified in the Thai Ministry of Public Health Notifications No. 355 (2013) and No. 416 (2020), demonstrating that the products met the required microbiological quality standards at the time of analysis.

#### 3.4.3. Sensory Properties

Sensory acceptability scores of the original‐ and paprika‐flavored extruded snacks are presented in Table 6. Both samples received favorable ratings from the panelists, with mean scores above 6.0 for all evaluated attributes on the 9‐point hedonic scale, indicative of good overall acceptance. The overall liking scores were 7.62 for the original‐flavored snack and 7.68 for the paprika‐flavored snack, suggesting that the addition of paprika seasoning did not negatively affect consumer perception.

**Table 6:** Sensory acceptability scores of original‐ and paprika‐flavored extruded snacks evaluated using a 9‐point hedonic scale.

Sample	Liking score								
	Color	Size	Shape	Odor	Crispiness	Hardness	Sweetness	Saltiness	Overall acceptability
Original flavor	7.22	6.10	7.88	7.10	7.92	7.44	6.96	7.12	7.62
Paprika flavor	6.94	7.70	7.82	6.80	8.00	7.72	7.18	7.34	7.68

*Note:* Sensory acceptability was evaluated using a 9‐point hedonic scale, where 1 = *dislike extremely*, 2 = *dislike very much*, 3 = *dislike moderately*, 4 = *dislike slightly*, 5 = *neither like nor dislike*, 6 = *like slightly*, 7 = *like moderately*, 8 = *like very much*, and 9 = *like extremely*.

Appearance attributes are important determinants of consumer acceptance, as they form the first impression of food quality [48]. Both products obtained relatively high scores for color, size, and shape. Texture attributes, particularly crispiness and hardness, were also highly rated, with respective crispiness scores of 7.92 and 8.00, respectively. Crispiness is a key quality attribute of extruded snack products and is closely related to the porous structure formed during extrusion [49]. Flavor‐related attributes, including odor, sweetness, and saltiness, were generally well accepted, with slightly higher sweetness and saltiness scores observed for the paprika‐flavored sample, likely due to the seasoning formulation. Seasonings are widely used to enhance the sensory appeal and consumer acceptance of snack products [28]. Overall, both products were well accepted by the panelists, suggesting that flavor variation can diversify product options without compromising sensory quality.

#### 3.4.4. Nutritional Properties

The nutritional composition of the original‐ and paprika‐flavored crispy fried shrimp legs snacks on a per‐serving basis (50 g) is presented in Figures 4 and 5. Both formulations provided 200 kcal per serving and contained 3 g of total fat (5% Thai RDI), including 1 g of saturated fat (5% Thai RDI), indicating a relatively low fat content despite the incorporation of fried shrimp leg powder. The cholesterol content ranged from 20 to 25 mg per serving (7%–8% Thai RDI), reflecting the natural cholesterol present in shrimp‐derived ingredients.

**Figure 4:**
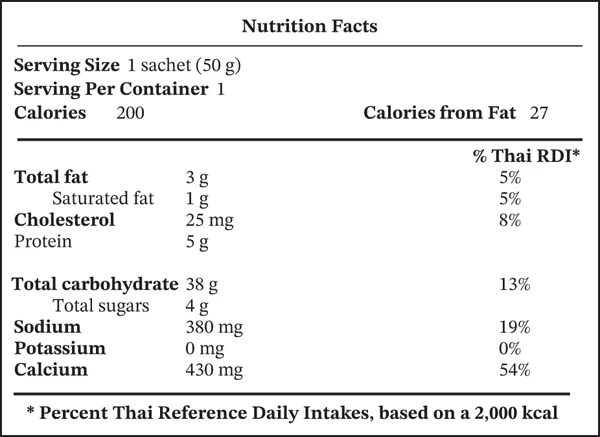
Nutrition facts of the crispy fried shrimp leg extruded snack (original flavor).

**Figure 5:**
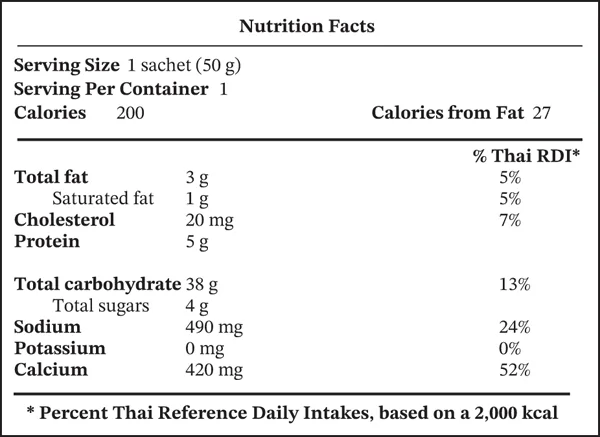
Nutrition facts of the crispy fried shrimp leg extruded snack (paprika flavor).

Each serving supplied 5 g of protein and 38 g of total carbohydrates (13% Thai RDI), including 4 g of total sugars. The protein content demonstrates that crispy fried shrimp leg powder can contribute to the nutritional value of cereal‐based extruded snacks while maintaining acceptable fat and energy contents. Sodium contents were 380 and 490 mg per serving (19 and 24% Thai RDI) for the original‐ and paprika‐flavored products, respectively, with the higher sodium level in the paprika formulation attributable to the seasoning ingredients. Potassium was not detected in either formulation.

Overall, the results indicate that the developed snacks provide moderate energy while containing relatively high levels of protein and calcium compared with conventional snack products. Together with their favorable chemical composition, satisfactory microbiological quality, and good sensory acceptability, these findings suggest that the products may represent a nutritionally beneficial and safe alternative for snack consumption.

## 4. Conclusions

This study demonstrated the potential of crispy fried shrimp legs, a shrimp processing by‐product, as a functional ingredient utilized in extruded snack products. The pretreatment process produced shrimp leg powder with high protein and calcium contents, indicating its value as a nutritionally rich raw material. Incorporation of shrimp leg powder influenced expansion and texture due to interactions between proteins, minerals, chitin, and the starch matrix during extrusion.

The optimal formulation containing 15% shrimp leg powder processed at 400 rpm screw speed and 120°C barrel temperature, and it produced snacks with desirable expansion, low density, and a crispy texture. The developed products also exhibited low moisture and water activity, favorable nutritional profiles, microbiological safety in accordance with Thai food standards and good sensory acceptability.

These findings highlight the potential of shrimp leg by‐products as a sustainable ingredient for developing value‐added, nutritionally enhanced extruded snack foods.

## Author Contributions

Riantong Singanusong: conceptualization, methodology, funding acquisition, project administration, resources, supervision, and writing—original draft preparation. Kanyarat Banyam: investigation, formal analysis, and writing—review and editing. Jiratchaya Judphol: writing—review and editing. Saowaluk Rungchang: methodology, supervision, and writing–review and editing.

## Funding

This study was supported by the Naresuan University (NU), and National Science, Research and Innovation Fund (NSRF) (R2568B051).

## Conflicts of Interest

The authors declare no conflicts of interest.

## Data Availability

The data are available on request from the authors.
